# Papillon-Lefèvre Syndrome: A Series of Six Cases in the Same Family

**DOI:** 10.5402/2012/139104

**Published:** 2012-12-03

**Authors:** Ali Kord Valeshabad, Abdolmotaleb Mazidi, Reza Kord Valeshabad, Elham Imani, Hadi Kord, Mohammad Koohkan, Zrynal Sayinar, Khalil Al-Talib

**Affiliations:** ^1^Division of Gastroenterology and Hepatology, School of Medicine, The Johns Hopkins University, 1800 Orleans Street, Sheikh Zayed Building, Room 7125 B, Baltimore, MD 21287, USA; ^2^Aliabad-e-Katoul Hygiene Center, Golestan University of Medical Sciences, Gorgan 4934174515, Iran; ^3^Zainaldin Martyr Research Center, Gorgan University of Agricultural Sciences and Natural Resources, P.O. Box 15739-49138, Gorgan, Iran; ^4^Department of Dermatology, Golestan University of Medical Sciences, Gorgan 4934174515, Iran; ^5^Department of Dermatology, Tehran University of Medical Sciences, P.O. Box 14155-6447, Tehran, Iran

## Abstract

Papillon-Lefèvre syndrome (PLS) is a rare, autosomal recessive heterogeneous disorder, which is characterized by palmoplantar hyperkeratosis, early loss of primary and permanent teeth, and associated calcification of the dura mater. Herein we described six cases of PLS in the same family. In this series, six cases (two females and four males) with the mean age of 15.6 ± 10.4 years were recruited. Palmoplantar hyperkeratosis was detected in all of the cases, leading to a difficult and painful walking in two cases due to lesions on the soles. Skin lesions were sharply distinct from adjacent normal skin in all cases. Other skin lesions were located in the external malleolus (5/6), knee (4/6), elbow (4/6), toe and dorsal fingers (3/6), and the thighs (2/6). In three cases, all permanent teeth were exfoliated. In three others, no primary teeth remained. Severe gingivitis was observed in three patients. Radiologic study confirmed alveolar bone destruction in five cases. Delayed diagnosis and insufficient treatment of PLS patients can affect patient's life of by causing edentulism at a young age and may impose PLS patients to increased risk of social, psychological, and economical burdens.

## 1. Introduction

Papillon-Lefèvre syndrome (PLS) is a rare, autosomal recessive heterogeneous disorder, which was first described by Papillon Lefèvre in 1924 [1]. PLS is characterized by palmoplantar hyperkeratosis, early loss of primary and permanent teeth, and associated calcification of the dura mater [2, 3]. The incidence rate of PLS is between one to four persons per million with no gender prominence. Consanguineous marriage is determined in 20 to 40% of patients with PLS [4].

Palmoplantar lesions are usually presented during the time of tooth eruption between the ages of six months to three years. The initial lesions can occasionally be mistaken for eczema and can rapidly progress in most cases. Other regions, including the eyelids, cheeks, knees, elbows, thighs, labial commissures, external malleolus, toes, and dorsal fingers may also be involved [1, 5].

Periodontal involvement is typically presented immediately after tooth eruption, accompanied by severe gingival inflammation, leading to exfoliation of primary teeth by age of four to five [1, 5]. Gingival inflammation is typically revealed after primary teeth exfoliation and is recurrent as the permanent teeth erupt. Around the age of fifteen, the majority of the permanent teeth are lost [6]. There is a dramatic alveolar bone resorption, which leads to a “floating-in-air appearance” in the dental imaging [7]. Most of the previous studies reported one to three cases of Papillon-Lefèvre syndrome [8–10]. In this series, we described six cases of PLS within the same family.

## 2. Cases

This case-series study was conducted during routine patient visits at one of the rural centers of Aliabade-e-Katoul Hygiene Center in the Golestan Province, Iran. The study protocol was approved by the ethical committee at the Golestan University of Medical Sciences. All participants signed informed consent forms prior to their enrollment. First patient (case no. 1) presented with complaint of chronic eczema at a primary health center. Her family history showed similar problems in some other members of her family. Other cases were invited for further assessment. Patients underwent a detailed history taking and a physical examination by a general practitioner. Based on the current referral policy of the Ministry of Hygiene and Health accessible at hygiene centers severe cases were referred to the dermatologist and dentist. All cases initially underwent laboratory hematological and biochemistry assessments cell blood counting, liver function tests, alkaline phosphatase, and urine analysis. Patients were visited weekly to assess the improvement of their symptoms by the same physician.

## 3. Results

In this series, six cases (two females and four males) with PLS were recruited. The mean age was 15.6 ± 10.4 years. In fifty percent of the cases, consanguinity was confirmed in parents (first degree cousins). Pregnancy and delivery were normal in all cases. All of the cases were found within the same family (first- or second- degree relatives), and all came from low socioeconomic backgrounds. Patients' demographic and clinical data have been summarized in Table 1.

**Figure 1:**
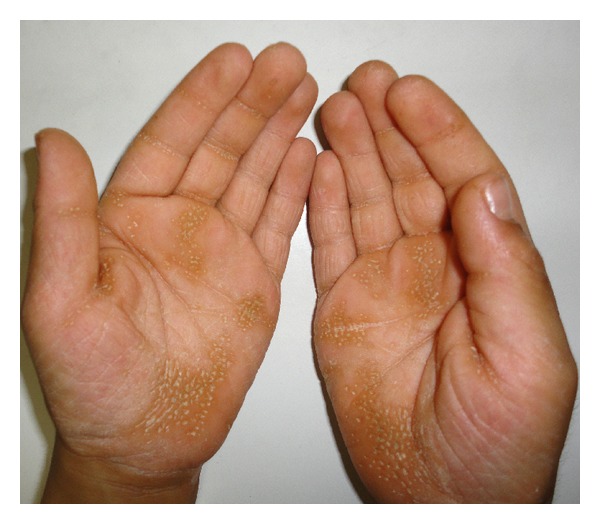
Palmar hyperkeratosis in case 5.

**Figure 2:**
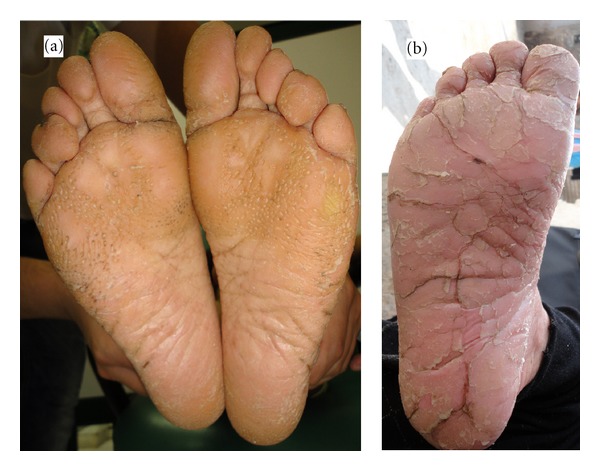
Plantar hyperkeratosis in (a) case 5 and (b) case 1.

**Figure 3:**
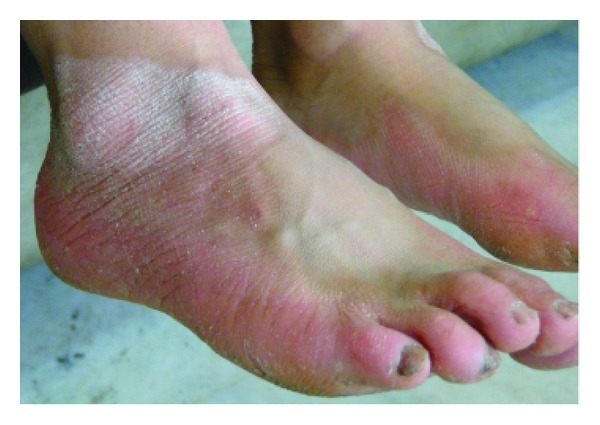
Involvement of external malleolus in case 5.

**Figure 4:**
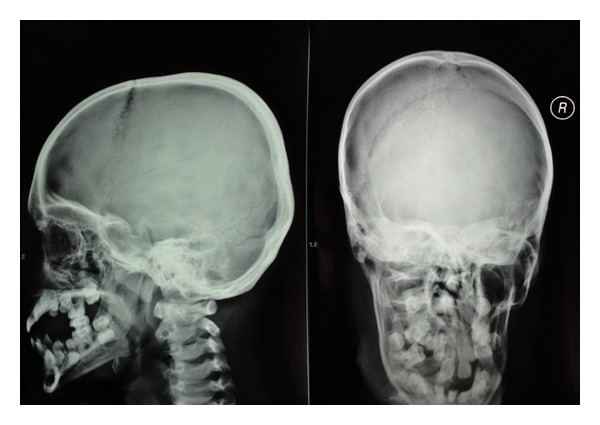
Cephalogram in case 5 showed no calcification. Alveolar bone destruction and severe periodontal destruction can also be seen in this figure.

Skin lesions had sharp margin and were distinct from adjacent normal skin in all cases. Palmoplantar hyperkeratosis was detected in all of the cases, leading to painful walking in two cases due to lesions on the soles. Figures 1 and 2 show palmoplantar hyperkeratosis in two cases. Other skin lesions were located in the external malleolus (5/6), knee (4/6), elbow (4/6), toe and dorsal fingers (3/6), and the thighs (2/6). Skin lesions in external malleolus have been shown in Figure 3. As it is shown in Figure 4 no signs of intracranial calcification were found from a lateral cephalogram X-ray of all patients. All patients with age greater than seven years old had symptoms of depression, including hopelessness, aimlessness, social phobia, and a fear of communicating with people outside their family.

Oral hygiene was poor in all the cases. In three cases, all permanent teeth were exfoliated. In three others, no primary teeth remained. Severe gingivitis was observed in three of the cases. A radiologic study confirmed alveolar bone destruction in five cases. None of the patients were diagnosed with PLS previously, and all of them had received only local and topical corticosteroids, neither of which was successful. Due to financial issues, patients had no compliance with previous treatments. Patients were referred to a dentist for further treatment but were unable to receive care due to the financial issues.

## 4. Discussion

While extremely rare, PLS is associated with life-long psychological and social impacts on growing children. Patients diagnosed with PLS suffer from its adverse effects throughout adolescence. All patients with age greater than seven years old had symptoms of depression, including hopelessness, aimlessness, social phobia, and a fear of communicating with people outside their family. 

The exact etiology of PLS is still obscure; however, microbiologic, immunologic, and genetic factors have all been linked to the development of the syndrome. *Actinobacillus actinomycetemcomitans* was reported to have a significant role in the progression of periodontal involvements [11]. Other microbial agents including* Porphyromonas gingivalis*, *Fusobacterium nucleatum*, and *Treponema denticola* have also been suggested to have causal effects [12].

Recent investigations have illustrated that, like Haim-Munk syndrome, PLS can be caused by defects in cathepsin C gene located on the 11q14-q21 region of the chromosome [12, 13]. Cathepsin C has roles in T-cell activation, as well as skin maintenance [13]. PLS differs from Haim-Munk syndrome in symptoms such as arachnodactyly, acroosteolysis, and onychogryphosis, which are only present in Haim-Munk syndrome [12, 13]. None of our cases demonstrated such symptoms, and thus, Haim-Munk syndrome was rolled out.

Neutrophil and T-cell dysfunctions have been suggested as immunological features of PLS [9]. Chemotactic and phagocytic function of neutrophils is decreased in PLS, and it is associated with a diminished phytohemagglutinin response by T-cell lymphocytes [9, 12]. In consistence with previous reports, consanguineous marriage was found in fifty percent of our patients implying the genetic basis for the disease [14, 15]. Due to low socioeconomic status, genetic testing was not performed to identify responsible mutations.

Various treatment modalities have been proposed for PLS including early extraction of primary teeth, systemic and local antibiotic treatment, and synthetic retinoids [4]. Without treatment, PLS patients could potentially be rendered edentulous in very young ages. This instills a social phobia within the patients, increasing the fear to communicate with people outside their family. Our first case had referred to both dermatologists and dentists frequently, but diagnosis was missed and was mistreated as eczema. It is interesting to note that these series were discovered by a family practitioner in the first line of health referral network in our country. This implies the importance of basic intervention and the key role of primary physicians who initially connect the patients into the health network including family practitioner and pediatric dentist. If these cases had been diagnosed earlier, patients might not have suffered such financial, psychological, and social burdens. To our best knowledge, this is the largest series of PLS cases in the same family. Genetic assessment of such large familial series could be of prime importance; however, due to financial issues we did not perform genetic evaluations in this study. Regional or international support is required to perform genetic assessment and to provide appropriate treatment in this family. We tried to perform orthopantomogram (OPG) in cases with primary teeth (cases 4, 5, and 6), but due to their age and mental retardation they had no cooperation, and we failed to prepare an appropriate OPG.

## 5. Conclusion

Delayed diagnosis and insufficient treatment of PLS patients can affect patient's life by early edentulism and will impose a great risk of social, psychological, and economical burdens on patients. Further studies are required to discover a genetic basis and to establish appropriate treatment modalities.

## Figures and Tables

**Table 1:** Patients' demographic and clinical data.

Variables		Cases					
		1	2	3	4	5	6
Gender		F	M	M	F	M	M
Age		30	21	23	9	7	4

Periodontal manifestations	Gingivitis	−	+	+	+	+	+
	Primary teeth loss	+	+	+	+	+	+
	Permanent teeth loss	+	+	+	−	−	−
	Alveolar bone resorption	+	+	+	+	+	−
	Halitosis	+	+	+	−	−	−

Skin manifestations	Palmoplantar hyperkeratosis	+	+	+	+	+	+
	Eyelids	−	−	−	+	−	+
	Cheeks	−	−	+	−	+	−
	Elbows	+	+	−	+	+	−
	Thighs	−	−	+	−	+	−
	Knees	+	+	−	+	+	−
	Toes	+	+	−	+	−	−
	External malleolus	+	+	+	−	+	+
	Dorsal fingers	+	−	−	+	−	+
	Labial commissures	−	−	−	+	−	−

Mental retardation		+	+	−	+	+	−
